# Is noma an infectious disease? Is it transmissible?

**DOI:** 10.1186/1753-6561-5-S6-P251

**Published:** 2011-06-29

**Authors:** A Gayet-Ageron, D Baratti-Mayer, D Courvoisier, D Pittet, GESNOMA

**Affiliations:** 1 Infection Control Program, Geneva, Switzerland; 2 University Hospitals Of Geneva, Geneva, Switzerland; 3 Division of Clinical Epidemiology, University Hospitals of Geneva, Geneva, Switzerland

## Introduction / objectives

Noma is a devastating facial necrosis affecting young children in developing countries. The causative agents are not well identified, in particular the association of noma with specific microbiological agents and the risk for transmission.

## Methods

Prospective matched case-control study conducted in Zinder, Niger, between September 2001 and October 2006. Epidemiological, socio-behavioural, and biological determinants were collected through interviews with children and their representatives and during clinical examinations. Conditional logistic regression was applied.

## Results

A total of 82 acute noma cases and 327 controls were recruited. The noma epidemic curve declined during the study-period (*P*=0.04) with no seasonal effect (*P*=0.74). There was no intra-family case, but an older sibling rank was associated with a higher odds of developing noma (OR>=3^rd^ position 3.51; 95%CI: 1.57-7.85). Noma was also associated with severe wasting (OR 7.79; 3.89-15.57), severe stunting (OR 5.22; 2.73-9.97), a higher number of past pregnancies in the mother (OR 1.19 for each additional child; 1.08-1.32), the presence for any other disease within the last 3 months (OR 3.52; 1.89-6.54) or family posses no chicken at home, as aproxy for poverty (OR 2.53; 1.32-4.82). No association was observed between noma and serological status to various viruses (EBV, VZV, HSV, CMV, Morbillivirus). Definitive microbiological data will be available at time of the meeting.

## Conclusion

Noma is linked with poor general health status leading to a higher risk to develop opportunistic illnesses, which precipitates the occurrence of devastating facial lesions. No epidemiological evidence was shown for cross-transmission in this cohort- the largest reported to the best of our knowledge.

## Disclosure of interest

None declared.

